# ﻿Morphological and phylogenetic analyses reveal three new species and one new record of *Tubeufia* (Tubeufiales, Tubeufiaceae) from southern China

**DOI:** 10.3897/mycokeys.99.107606

**Published:** 2023-08-14

**Authors:** Jian Ma, Li-Juan Zhang, Saranyaphat Boonmee, Xing-Juan Xiao, Ning-Guo Liu, Yuan-Pin Xiao, Zong-Long Luo, Yong-Zhong Lu

**Affiliations:** 1 School of Food and Pharmaceutical Engineering, Guizhou Institute of Technology, Guiyang 550003, China; 2 Center of Excellence in Fungal Research, Mae Fah Luang University, Chiang Rai 57100, Thailand; 3 School of Science, Mae Fah Luang University, Chiang Rai, 57100, Thailand; 4 College of Agriculture and Biological Science, Dali University, Dali 671003, China

**Keywords:** asexual morph, new taxa, phylogeny, taxonomy

## Abstract

During an investigation of helicosporous fungi in China, a total of seven helicosporous hyphomycetes were obtained from decaying wood in the southern region of the country. Based on phylogenetic analyses using a combined LSU, ITS, *tef1α*, and *rpb2* sequence matrix, in conjunction with morphological comparisons, these taxa were classified within *Tubeufia* (Tubeufiaceae, Tubeufiales) and were recognized as three new species, *viz. Tubeufiaguttulata*, *T.hainanensis*, and *T.muriformis*, as well as one new distribution record, *viz. T.cocois*. Evidence for these new taxa and the new record, descriptions, illustrations, notes, and phylogenetic evidence are provided for the newly collected helicosporous species.

## ﻿Introduction

*Tubeufia* was introduced by Penzig and Saccardo (1897) with the type species *T.javanica* and two other species (*T.anceps* and *T.coronata*). *Tubeufia* species are widely distributed in tropical and temperate regions, including freshwater and terrestrial habitats, occurring primarily on well-rotted wood (Barr 1979; Rossman 1987; Ho et al. 2002; Tsui and Berbee 2006; Tsui et al. 2007; Zhao et al. 2007; Boonmee et al. 2011, 2014; Chaiwan et al. 2017; Dai et al. 2017; Doilom et al. 2017; Lu et al. 2017, 2018a, 2018b, 2022, 2023; Tian et al. 2022). There are currently 56 accepted species in *Tubeufia*, as listed in Table 1. These species exhibit a wide range of morphological characteristics in both their sexual and asexual morphs. For example, although *Tubeufiabambusicola*, *T.javanica*, and *T.latispora* shared a close phylogenetic affinity, they possess distinct morphological features. *Tubeufiabambusicola* and *T.latispora* possess black ascomata that are densely covered with long, flexuous setae. These ascomata contain sessile asci, each containing eight fusiform ascospores. In contrast, *T.javanica* has white ascomata without seta and short pedicellate asci with eight filiform ascospores (Boonmee et al. 2014; Lu et al. 2017, 2018b). The asexual morphs of *Tubeufia* encompass both helicosporous and dictyosporous hyphomycetes. The helicosporous asexual morphs of *Tubeufia* display diverse shapes, with certain species exhibiting abundant, elongated conidiophores, while others possess extremely short conidiophores. For instance, *T.abundata* is characterized by abundant, branched, and long conidiophores, *T.arctata* exhibits rarely unbranched, short conidiophores, and *T.machaerinae* does not possess conidiophores (Lu et al. 2018b).

**Table 1.** Checklist of accepted *Tubeufia* species.

No.	Species	Distribution	Habitat	Molecular data	Reference
1	*T.abundata*	Thailand	Freshwater	Available	Lu et al. 2018b
2	*T.acaciae*	India	Terrestrial	Not available	Tilak and Kale 1969
3	*T.aciculospora*	Japan	Terrestrial	Not available	Katsumoto and Harada 1979
4	*T.aquatica*	China, Thailand	Freshwater	Available	Luo et al. 2017; Lu et al. 2018b
5	*T.bambusicola*	Thailand	Terrestrial	Available	Lu et al. 2018b
6	*T.brevis*	Thailand	Freshwater	Available	Lu et al. 2018b
7	*T.brevispina*	USA	Terrestrial	Not available	Barr and Rogerson 1983; Crane et al. 1998
8	*T.brunnea*	Thailand	Freshwater	Available	Lu et al. 2018b
9	*T.chiangmaiensis*	Thailand	Terrestrial	Available	Boonmee et al. 2014
10	*T.chlamydospora*	Thailand	Freshwater	Available	Lu et al. 2018b
11	*T.claspisphaeria*	China	Freshwater	Not available	Kodsueb et al. 2004
12	*T.cocois*	Thailand	Freshwater/ Terrestrial	Available	Tian et al. 2022
13	*T.cylindrothecia*	Thailand, USA	Freshwater	Available	Luo et al. 2017
14	*T.dactylariae*	China	Terrestrial	Not available	Chang 2003
15	*T.dentophora*	China	Terrestrial	Not available	Lu et al. 2018b
16	*T.dictyospora*	Thailand	Freshwater/ Terrestrial	Available	Lu et al. 2018b
17	*T.eccentrica*	China	Freshwater	Available	Lu et al. 2018b
18	*T.entadae*	Thailand	Terrestrial	Available	Jayasiri et al. 2019
19	*T.eugeniae*	India	Terrestrial	Not available	Pande 2008
20	*T.fangchengensis*	China	Freshwater	Available	Lu et al. 2018b
21	*T.filiformis*	Thailand	Freshwater	Available	Lu et al. 2017
22	*T.freycinetiae*	Thailand	Terrestrial	Available	Tibpromma et al. 2018
23	*T.garugae*	India	Terrestrial	Not available	Pande 2008
24	*T.geniculata*	China	Freshwater	Available	Lu et al. 2018b
25	*T.guangxiensis*	China	Freshwater	Available	Chaiwan et al. 2017
26	*T.hechiensis*	China	Freshwater	Available	Lu et al. 2018b
27	*T.hyalospora*	Thailand	Freshwater	Available	Hyde et al. 2016
28	*T.inaequalis*	Thailand	Freshwater	Available	Lu et al. 2018b
29	*T.javanica*	Thailand	Terrestrial	Available	Boonmee et al. 2014
30	*T.krabiensis*	Thailand	Freshwater	Available	Lu et al. 2018b
31	*T.latispora*	Thailand	Freshwater	Available	Lu et al. 2017
32	*T.laxispora*	Thailand	Freshwater	Available	Lu et al. 2017
33	*T.lilliputea*	Australia, China, India, Japan, USA	Terrestrial	Available	Lu et al. 2018b
34	*T.liyui*	China	Freshwater	Available	Lu et al. 2023
35	*T.longihelicospora*	China, Thailand	Freshwater	Available	Boonmee et al. 2021; Tian et al. 2022
36	*T.longiseta*	Thailand	Terrestrial	Available	Dai et al. 2017
37	*T.machaerinae*	China, USA	Freshwater/ Terrestrial	Available	Lu et al. 2018b
38	*T.mackenziei*	Thailand	Freshwater	Available	Lu et al. 2017
39	*T.nigroseptum*	China	Freshwater	Available	Li et al. 2022
40	*T.minuta*	Denmark, Sweden	Terrestrial	Not available	Munk 1966
41	*T.pachythrix*	Brazil	Terrestrial	Not available	Rossman 1979
42	*T.pandanicola*	Thailand	Terrestrial	Available	Tibpromma et al. 2018
43	*T.parvispora*	Thailand	Terrestrial	Available	Tibpromma et al. 2018
44	*T.parvula*	Britain, Sweden	Terrestrial	Not available	Dennis 1975
45	*T.roseohelicospora*	Thailand	Freshwater	Available	Hyde et al. 2016
46	*T.rubra*	Thailand	Freshwater	Available	Lu et al. 2018b
47	*T.sahyadriensis*	India	Terrestrial	Available	Rajeshkumar et al. 2019
48	*T.sessilis*	Thailand	Terrestrial	Available	Lu et al. 2018b
49	*T.silentvalleyensis*	India	Terrestrial	Not available	Pande 2008
50	*T.sympodihylospora*	China	Freshwater	Available	Lu et al. 2018b
51	*T.sympodilaxispora*	China	Freshwater	Available	Lu et al. 2018b
52	*T.sympodiophora*	China, Peru	Freshwater/ Terrestrial	Not available	Lu et al. 2018b
53	*T.taiwanensis*	China	Freshwater	Available	Lu et al. 2018b
54	*T.tectonae*	Thailand	Freshwater/ Terrestrial	Available	Doilom et al. 2017; Lu et al. 2018b
55	*T.tratensis*	Thailand	Freshwater	Available	Lu et al. 2018b
56	*T.xylophila*	China, India	Freshwater/ Terrestrial	Available	Lu et al. 2018b

The helicosporous taxa of *Tubeufia* represent a promising biological resource capable of producing novel bioactive secondary metabolites. Fan et al. (2019) discovered that *T.rubra* exhibited a significant antifungal impact against seven plant pathogenic fungi, *viz. Alternaria solani* (ZYB), *Botryosphaeriadothidea* (B12), *Fusariumgraminearum* (CM), *Magnaporthegrisea* (DWB), *Phoma* sp. (HGHM), *Phytophthoraparasitica* (HJB) and *Rhizoctorziasolani* (WB). The inhibition rate of *T.rubra* against these fungal pathogens exceeded 60%, indicating its potential as an effective antifungal agent. Fan et al. (2019) also discovered that the ethyl acetate extract from the fermentation broth of *T.machaerinae* inhibited the human cervical cancer cell line (HeLa) and human prostate cancer cell line (PC-3) by (98.92 ± 0.15%) and (97.86 ± 0.18%), respectively. According to Chen et al. (2020), both *T.hechiensis* and *T.rubra* exhibited the ability to reverse multidrug resistance in tumor cells. Zeng et al. (2022) discovered a newly isolated compound called Rubricin A from *T.rubra*, which demonstrated the capacity to inhibit the expression of P-glycoprotein (P-gp) and effectively reverse multidrug resistance in tumor cells. Qian et al. (2023) reported the isolation of two novel compounds, *viz.* Rubracin D and E, and sixteen known glyceroglycolipids from *T.rubra*. Rubracin D and E demonstrated significant multidrug resistance reversal activities on MCF-7/ADM, K562/ADM, and A549/ADM cell lines.

In this study, seven helicosporous taxa were collected from the southern provinces of Hainan and Guizhou in China. Based on morphological evidence and phylogenetic analyses, three novel species were introduced and designated as *Tubeufiaguttulata*, *T.hainanensis* and *T.muriformis*, and one new distribution record, *viz. T.cocois*. The new species are described with detailed morphological descriptions and illustrations.

## ﻿Materials and methods

### ﻿Sample collection, specimen examination, and isolation

From August 2021 to March 2022, decaying wood samples were collected from Hainan and Guizhou provinces in southern China. The collected information includes locations, dates, altitudes, latitudes, and longitude. Fresh specimens were placed in zip-lock bags and sterile, moist plastic boxes, and then incubated at room temperature for a period of two weeks. Using a stereomicroscope (SMZ-168, Nikon, Japan), the fungal colonies growing on decaying wood surfaces were carefully examined, observed, and documented through photography. Morphological features such as conidiophores, conidiogenous cells, and conidia were further captured using an ECLIPSE Ni compound microscope (Nikon, Tokyo, Japan) in conjunction with a Canon 90D digital camera. Measurements were taken with the Tarosoft (R) Image Frame Workprogram. Photoplates were developed with Adobe PhotoShop CC 2019 (Adobe Systems, USA).

According to the method described by Senanayake et al. (2020), helicosporous strains were isolated and purified on water agar (WA), and germinated conidia were aseptically transferred to fresh potato dextrose agar (PDA) plates. Mycelium was cultured on Potato Dextrose Agar (PDA) and incubated at a temperature of 25 °C for a period of 6–7 weeks. During this time, various morphological characteristics including color, shape, and size were carefully observed and recorded. Subsequently, dried specimens of the fungal cultures were deposited in the Herbarium of Kunming Institute of Botany, Chinese Academy of Sciences (Herb. HKAS), Kunming, China, and the Herbarium of Guizhou Academy of Agriculture Sciences (Herb. GZAAS), Guiyang, China. Cultures were deposited in the China General Microbiological Culture Collection Center (CGMCC; https://www.cgmcc.net/english/), Beijing, China, and the Guizhou Culture Collection, China (GZCC), Guiyang, China for future reference and scientific documentation. Faces of fungi (FOF; https://www.facesoffungi.org) numbers were obtained in accordance with Jayasiri et al. (2015) and MycoBank numbers of the new species were registered in the MycoBank database (https://www.mycobank.org/).

### ﻿DNA extraction, PCR amplification, and sequencing

Fresh fungal hyphae were gently scraped and transferred to a 1.5-mL microcentrifuge tube using a sterile toothpick. Genomic DNA was extracted using the Biospin Fungus Genomic DNA Extraction Kit (BioFlux, China) and following the manufacturer’s protocol for DNA extraction. Specific forward and reverse primers, namely ITS5/ITS4, LR0R/LR5, fRPB2-5F/fRPB2-7cR, and EF1-983F/EF1-2218R, were employed to amplify the internal transcribed spacer (ITS, White et al. 1990), large ribosomal subunit (LSU, Vilgalys and Hester 1990), and RNA polymerase II second-largest subunit (*rpb2*, Liu et al. 1999) and translation elongation factor 1-α gene (*tef1α*, Rehner and Buckley 2005) sequence fragments, respectively. The polymerase chain reaction (PCR) conditions employed were in accordance with the reaction conditions outlined in the publications by Lu et al. (2017, 2018a). PCR amplification reactions were conducted in a 50-μL reaction volume containing 44 μL of 1.1× T3 Supper PCR Mix (Qingke Biotech, China), 2 μL of forward and reverse primers, and 2 μL of DNA template. The products were detected by 1% agarose gel electrophoresis following PCR amplification, with primers and PCR product sequencing provided by Beijing Qingke Biotechnology Co., Ltd.

### ﻿Phylogenetic analyses

The original sequences were examined with BioEdit v 7.0.5.3 (Hall 1999) and assembled with SeqMan v. 7.0.0 (DNASTAR, Madison, WI, USA; Swindell and Plasterer 1997). The taxa used in this study (Table 2) were downloaded based on high levels of identity (> 90%) and selected outgroups (https://blast.ncbi.nlm.nih.gov/Blast.cgi). The polygenic dataset was aligned by MAFFT v.7.473 (https://mafft.cbrc.jp/alignment/server/, Katoh and Standley 2013; Katoh et al. 2019). The data were trimmed using trimAl.v1.2rev59 software (Capella-Gutiérrez et al. 2009). The multigenic sequences (LSU-ITS-*tef1α*-*rpb2*) were merged using SequenceMatrix-Windows-1.7.8 software (Vaidya et al. 2011). The aligned Fasta file was converted to Nexus format file for Bayesian inference (BI) analyses using AliView v. 1.27 (Daniel et al. 2010).

**Table 2.** Taxa used in this study and their GenBank accession numbers of DNA sequences.

Taxon	Strain	GenBank Accessions			
		ITS	LSU	*tef1α*	*rpb2*
*Acanthohelicosporaaurea*	GZCC 16-0060	KY321323	KY321326	KY792600	MF589911
*A.guianensis*	UAMH 1699	AY916479	AY856891	–	–
*T.abundata*	MFLUCC 17-2024^T^	MH558769	MH558894	MH550961	MH551095
*T.aquatica*	MFLUCC 16-1249^T^	KY320522	KY320539	KY320556	MH551142
*T.aquatica*	DLUCC 0574	–	KY320538	KY320555	MH551141
*T.aquatica*	MFLUCC 17-1794	MH558770	MH558895	MH550962	MH551096
*T.bambusicola*	MFLUCC 17-1803^T^	MH558771	MH558896	MH550963	MH551097
*T.brevis*	MFLUCC 17-1799^T^	MH558772	MH558897	MH550964	MH551098
*T.brunnea*	MFLUCC 17-2022^T^	MH558773	MH558898	MH550965	MH551099
*T.chiangmaiensis*	MFLUCC 17-1801	MH558774	MH558899	MH550966	MH551100
*T.chiangmaiensis*	MFLUCC 11-0514^T^	KF301530	KF301538	KF301557	–
*T.chlamydospora*	MFLUCC 16-0223^T^	MH558775	MH558900	MH550967	MH551101
*T.cocois*	MFLUCC 22–0001^T^	OM102541	OL985957	OM355486	OM355491
***T.cocois***	**GZCC 22-2038**	**OR030844**	**OR030837**	**OR046681**	–
*T.cylindrothecia*	BCC 3559	–	AY849965	–	–
*T.cylindrothecia*	BCC 3585	AY916482	AY856908	–	–
*T.cylindrothecia*	DLUCC 0572	KY320520	KY320537	KY320554	–
*T.cylindrothecia*	MFLUCC 16-1253	KY320519	KY320536	KY320553	–
*T.cylindrothecia*	MFLUCC 16-1283	KY320518	KY320535	KY320552	MH551143
*T.cylindrothecia*	MFLUCC 17-1792	MH558776	MH558901	MH550968	MH551102
*T.dictyospora*	MFLUCC 17-1805^T^	MH558778	MH558903	MH550970	MH551104
*T.dictyospora*	MFLUCC 16-0220	MH558777	MH558902	MH550969	MH551103
*T.eccentrica*	GZCC 16-0048	MH558780	MH558905	MH550972	MH551106
*T.eccentrica*	GZCC 16-0084	MH558781	MH558906	MH550973	MH551107
*T.eccentrica*	MFLUCC 17-1524^T^	MH558782	MH558907	MH550974	MH551108
*T.eccentrica*	GZCC 16-0035	MH558779	MH558904	MH550971	MH551105
*T.entadae*	MFLU 18-2102	MK347727	MK347943	–	–
*T.fangchengensis*	MFLUCC 17-0047^T^	MH558783	MH558908	MH550975	MH551109
*T.filiformis*	MFLUCC 16-1128^T^	–	KY092407	KY117028	MF535284
*T.filiformis*	MFLUCC 16-1135	KY092416	KY092411	KY117032	MF535285
*T.filiformis*	MFLUCC 16-0236	–	MH558938	MH550976	MH551110
*T.freycinetiae*	MFLUCC 16-0252^T^	MH275089	MH260323	MH412786	–
*T.geniculata*	BCRC FU30849^T^	LC335817	–	–	–
*T.geniculata*	NCYU U2-1B	LC335816	–	–	–
*T.guangxiensis*	GZCC 16-0054	MG012027	MG012020	MG012006	MG012013
*T.guangxiensis*	GZCC 16-0090	MG012029	MG012022	MG012008	MG012015
*T.guangxiensis*	GZCC 16-0091	MG012028	MG012021	MG012007	MG012014
*T.guangxiensis*	MFLUCC 17-0038	MG012026	MG012019	MG012005	MG012012
*T.guangxiensis*	MFLUCC 17-0045^T^	MG012025	MG012018	MG012004	MG012011
*T.guangxiensis*	MFLUCC 17-0046	MH558784	MH558909	MH550977	MH551111
*T.guangxiensis*	GZCC 16-0041	MG012030	MG012023	MG012009	MG012016
***T.guttulata***	**GZCC 23-0404^T^**	**OR030841**	**OR030834**	**OR046678**	**OR046684**
***T.guttulata***	**GZCC 23-0590**	**OR066413**	**OR066420**	**OR058859**	**OR058852**
***T.hainanensis***	**GZCC 22-2015^T^**	**OR030842**	**OR030835**	**OR046679**	**OR046685**
***T.hainanensis***	**GZCC 23-0589**	**OR066414**	**OR066421**	**OR058860**	**OR058853**
*T.hechiensis*	MFLUCC 17-0052^T^	MH558785	MH558910	MH550978	MH551112
*T.hyalospora*	MFLUCC 15-1250^T^	MH558786	MH558911	MH550979	–
*T.inaequalis*	GZCC 16-0079	MH558787	MH558912	MH550980	MH551113
*T.inaequalis*	GZCC 16-0087	MH558788	MH558913	MH550981	MH551114
*T.inaequalis*	MFLUCC 17-0053^T^	MH558789	MH558914	MH550982	MH551115
*T.inaequalis*	MFLUCC 17-1989	MH558790	MH558915	MH550983	MH551116
*T.inaequalis*	MFLUCC 17-1998	MH558791	MH558916	MH550984	MH551117
*T.inaequalis*	BCC 8808	AY916481	AY856910	–	–
*T.javanica*	MFLUCC 12-0545	KJ880034	KJ880036	KJ880037	–
*T.krabiensis*	MFLUCC 16-0228^T^	MH558792	MH558917	MH550985	MH551118
*T.latispora*	MFLUCC 16-0027^T^	KY092417	KY092412	KY117033	MH551119
*T.laxispora*	MFLUCC 16-0013	MH558793	MH558918	MH550986	MH551120
*T.laxispora*	MFLUCC 16-0219	KY092414	KY092409	KY117030	MF535286
*T.laxispora*	MFLUCC 16-0232^T^	KY092413	KY092408	KY117029	MF535287
*T.laxispora*	MFLUCC 17-2023	MH558794	MH558919	MH550987	MH551121
*T.lilliputea*	NBRC 32664	AY916483	AY856899	–	–
*T.liyui*	GZCC 22-2030^T^	OP888466	OP888465	–	–
*T.longihelicospora*	MFLUCC 21-0814	OM331690	OM331688	–	–
*T.longihelicospora*	MFLUCC 21-0815	OM331691	OM331705	–	–
*T.longihelicospora*	MFLUCC21-0151	OL606156	OL606149	OL964520	OL964526
*T.longihelicospora*	MFLUCC 16-0753^T^	NR_182938	–	–	–
*T.longiseta*	MFLUCC 15-0188^T^	KU940133	–	–	–
*T.machaerinae*	MFLUCC 17-0055^T^	MH558795	MH558920	MH550988	MH551122
*T.mackenziei*	MFLUCC 16-0222^T^	KY092415	KY092410	KY117031	MF535288
***T.muriformis***	**GZCC 22-2039^T^**	**OR030843**	**OR030836**	**OR046680**	**OR046686**
***T.muriformis***	**GZCC 23-0591**	**OR066415**	**OR066422**	**OR058861**	**OR058854**
*T.nigroseptum*	CGMCC 3.20430^T^	MZ092716	MZ853187	OM022002	OM022001
*T.pandanicola*	MFLUCC 16-0321^T^	MH275091	MH260325	–	–
*T.parvispora*	MFLUCC 17-1992	MH558796	MH558921	MH550989	MH551123
*T.parvispora*	MFLUCC 17-2003	MH558797	MH558922	MH550990	MH551124
*T.parvispora*	MFLUCC 17-2009	MH558798	MH558923	MH550991	MH551125
*T.roseohelicospora*	MFLUCC 16-0230	MH558799	MH558924	MH550992	MH551126
*T.roseohelicospora*	MFLUCC 17-1797	MH558800	MH558925	MH550993	MH551127
*T.roseohelicospora*	MFLUCC 15-1247^T^	KX454177	KX454178	–	MH551144
*T.rubra*	GZCC 16-0083^T^	MH558802	MH558927	MH550995	MH551129
*T.rubra*	GZCC 16-0081	MH558801	MH558926	MH550994	MH551128
*T.sahyadriensis*	NFCCI 4252/RAJ 99.1^T^	MH033849	MH033850	MH033851	–
*T.sahyadriensis*	NFCCI RAJ 99.2	MN393081	MN393082	MN393083	–
*T.sessilis*	MFLUCC 16-0021^T^	MH558803	–	MH550996	MH551130
*T.sympodihylospora*	GZCC 16-0051	MH558805	MH558929	MH550998	MH551132
*T.sympodihylospora*	MFLUCC 17-0044^T^	MH558806	MH558930	MH550999	MH551133
*T.sympodihylospora*	GZCC 16-0049	MH558804	MH558928	MH550997	MH551131
*T.sympodilaxispora*	BCC 3580	–	DQ296554	–	–
*T.sympodilaxispora*	GZCC 16-0058^T^	MH558807	MH558931	MH551000	MH551134
*T.sympodilaxispora*	MFLUCC 17-0048	MH558808	MH558932	MH551001	MH551135
*T.taiwanensis*	BCRC FU30844^T^	LC316605	–	–	–
*T.tectonae*	MFLUCC 16-0235	MH558809	MH558933	MH551002	MH551136
*T.tectonae*	MFLUCC 17-1985	MH558810	MH558934	MH551003	MH551137
*T.tectonae*	MFLUCC 12-0392^T^	KU144923	KU764706	KU872763	–
*T.tratensis*	MFLUCC 17-1993^T^	MH558811	MH558935	MH551004	MH551138
*T.xylophila*	MFLUCC 17-1520	MH558813	MH558937	MH551006	MH551140
*T.xylophila*	GZCC 16-0038	MH558812	MH558936	MH551005	MH551139
*Tubeufiaceae* sp.	BCC 3512	AY916484	AY856905	–	–
*Tubeufiaceae* sp.	BCC 3381	–	AY787932	–	–

Note: “^T^” denotes ex-type strain. Newly generated sequences are indicated in bold. “-” means no data available in GenBank.

Maximum likelihood (ML) analysis was carried out using the IQ Tree online website (http://iqtree.cibiv.univie.ac.at/), employing Bayesian Information Criteria (BIC) as the criterion for model selection, as described by Nguyen et al. (2015). The server automatically conducted tests to determine the appropriate substitution model for the analysis.

Bayesian inference (BI) analysis was conducted in MrBayes on XSEDE (3.2.7a) (Ronquist et al. 2012). The best-fit substitution model GRT + I +G was decided for LSU, ITS, *tef1α* and *rpb2* matrix by MrModeltest 2.3 under the Akaike Information Criterion (AIC) (Nylander et al. 2008). Four simultaneous Markov chains were run for 10,000,000 generations, and trees were sampled every 1000^th^ generations. Burn-in phase was set at 25% and the remaining trees were used for calculating posterior probabilities (PP).

Phylogenetic trees were visualized and edited using FigTree v. 1.4.4 and Adobe Illustrator CC 2019v. 23.1.0 (Adobe Systems, USA). In addition, Adobe PhotoShop CC 2019 (Adobe Systems, USA) was used to create the photo-plates.

## ﻿Phylogenetic results

**Figure 1.**
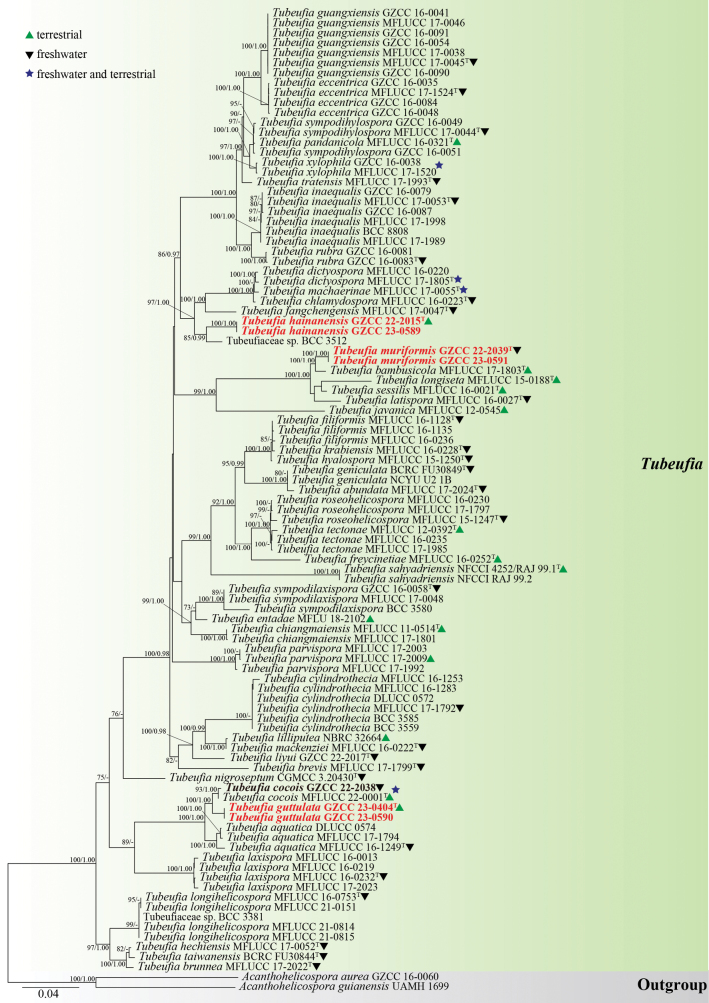
Phylogenetic tree generated from maximum likelihood (ML) analysis based on a combined LSU, ITS, *tef1α*, and *rpb2* sequence data. The bootstrap support values of ML are equal to or greater than 75%, and Bayesian posterior probabilities (PP) equal to or greater than 0.95 are given near the nodes as ML/PP, respectively. *Acanthohelicosporaaurea*GZCC 16–0060 and *A.guianensis* UAMH 1699 were used as outgroup taxa. The new species are indicated in red bold and newly generated sequences are indicated in black bold. “^T^” denotes ex-type strain. Symbols after generic names in *Tubeufia* indicate the habitats of taxa as explained in the phylogram.

The phylogenetic position of the newly isolated taxa was determined in this study using partial LSU-ITS-*tef1α*-*rpb2* nucleotide sequences. The concatenated sequence matrix consisted of LSU (1–845 bp), ITS (846–1440 bp), *tef1α* (1441–2352 bp), and *rpb2* (2353–3397 bp) for a total of 97 taxa, including two outgroup taxa, resulting in a matrix of 3,397 characters. Maximum likelihood (ML) and Bayesian inference (BI) analyses were conducted on the concatenated datasets of LSU, ITS, *tef1α*, and *rpb2*, both yielding similar tree topologies, and the ML tree is shown in Fig. 1.

Based on the multigene phylogenetic tree depicted in Fig. 1, our study revealed that the seven new collections belong to three distinct species within the genus *Tubeufia*. *Tubeufiaguttulata* is a significantly distinct lineage from *T.cocois* with 100% ML/1.00 PP supports. *T.hainanensis* establishes a sister lineage to Tubeufiaceae BCC 3512 with 85% ML/0.99 PP supports. *T.muriformis* can be distinguished from other related *Tubeufia* species by its distinct muriform conidia. Based on phylogenetic and morphological evidence, our new isolate, GZCC 22–2038, is recognized as *T.cocois*.

### ﻿Taxonomy

#### ﻿ Tubeufia guttulata

Taxon classificationFungiTubeufialesTubeufiaceae

J. Ma & Y.Z. Lu sp. nov.

31A04162-1662-5665-B2AC-F98EE1C37409

900504

Facesoffungi Number: FoF14265

Fig. 2

##### Etymology.

The epithet ‘‘*guttulata*’’ refers to the guttulate conidia of this taxon.

##### Holotype.

HKAS 128936

##### Description.

***Saprobic*** on decaying wood in a terrestrial habitat. ***Sexual morph*** Undetermined. ***Asexual morph*** Hyphomycetous, helicosporous. ***Colonies*** on natural substrate superficial, effuse, gregarious, white. ***Mycelium*** partly immersed, hyaline to pale brown, septate, branched hyphae, smooth, with masses of crowded, glistening conidia. ***Conidiophores*** macronematous, mononematous, flexuous, cylindrical, branched or unbranched, septate, 101–247 μm long, 5.5–8 μm wide (x̄ = 165 × 7 μm, n = 20), the lower part pale brown and the upper part hyaline, smooth-walled. ***Conidiogenous cells*** holoblastic, mono- to polyblastic, integrated, sympodial, intercalary or terminal, cylindrical, with a denticulate protrusion, truncate at apex after conidial secession, 9–16 μm long, 4–6 μm wide (x̄ = 12 × 5 μm, n = 25), hyaline to pale brown, smooth-walled. ***Conidia*** solitary, acropleurogenous, helicoid, rounded at tip, 25–34 μm diam and conidial filament 4–6 μm wide (x̄ = 29 × 5 μm, n = 30), 170–220 μm long (x̄ = 189 μm, n = 30), indistinctly septate, coiled 1^1^/_4_–2^1^/_4_ times, becoming uncoiled in water, guttulate, hyaline, smooth-walled.

**Figure 2.**
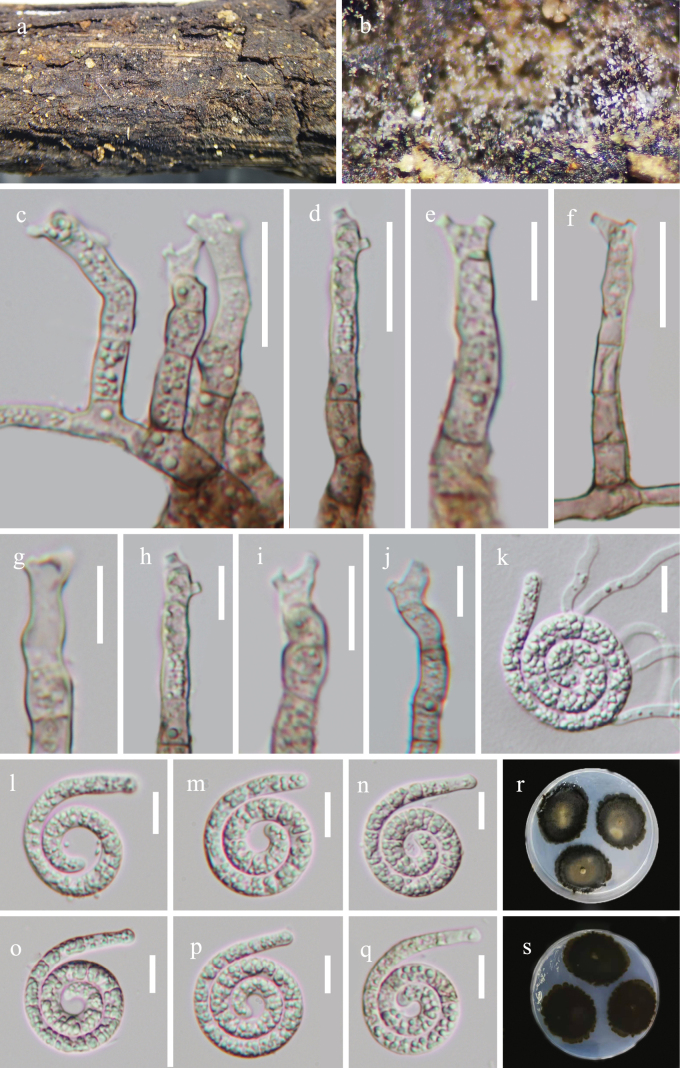
*Tubeufiaguttulata* (HKAS 128936, holotype) **a, b** colonies on the host surface **c–e** conidiophores, conidiogenous cells and conidia **f–i** conidiogenous cells bearing conidia **j** germinated conidium **k–p** conidia **q, r** colonies on PDA, **q** from above **r** from below. Scale bars: 50 μm (**c–e**); 20 μm (**h–p**); 10 μm (**f, g**).

##### Culture characteristics.

Conidia germinating on water agar and producing germ tubes within 8 h. Colonies on PDA circular with umbonate surface and undulate edge. Growth rate 21 mm diam in 42 days at 25 °C, with a brown center with pale brown edges on PDA.

##### Material examined.

China, Hainan Province, Wuzhishan City, Shui Man Town, Wuzhishan National Nature Reserve, 18°92′N, 109°63′E, on rotting wood in a terrestrial habitat, 26 December 2021, Jian Ma, WZS70 (HKAS 128936, holotype; GZAAS 23–0408, isotype), ex-type living cultures CGMCC, GZCC 23–0404; *Ibid*., WZS99 (GZAAS 23–0593, paratype), living culture GZCC 23–0590.

##### Notes.

*Tubeufiaguttulata* is a sister species to *T.cocois* with 100% ML/1.00 PP supports, however, the phylogenetic tree shows that they are distinct species. Morphologically, *Tubeufiaguttulata* differs from *T.cocois* in that it has longer conidiophores (101–247 μm vs. 38–123 μm) and larger conidia (170–220 μm vs. 116–136 μm). In addition, the helicoid conidia of *T.guttulata* become uncoiled in water, while *T.cocois* are coiled (Tian et al. 2022).

#### ﻿ Tubeufia hainanensis

Taxon classificationFungiTubeufialesTubeufiaceae

J. Ma & Y.Z. Lu sp. nov.

90623600-F6AF-5930-8E65-7076A4C54FC7

900505

Facesoffungi Number: FoF14266

Fig. 3

##### Etymology.

The epithet ‘‘*hainanensis*’’ refers to the collecting site.

##### Holotype.

HKAS 125884.

##### Description.

***Saprobic*** on decaying wood in moist ground. ***Sexual morph*** Undetermined. ***Asexual morph*** Hyphomycetous, helicosporous. ***Colonies*** on natural substrate superficial, effuse, gregarious, white. ***Mycelium*** superficial, partly immersed, hyaline to pale brown, septate, branched hyphae, smooth. ***Conidiophores*** macronematous, mononematous, straight to slightly flexuous, cylindrical, unbranched, septate, 44–56 μm long, 4–5 μm wide, pale brown to hyaline, smooth-walled. ***Conidiogenous cells*** holoblastic, mono- to polyblastic, integrated, sympodial, intercalary or terminal, cylindrical, with a denticulate protrusion, truncate at apex after conidial secession, 8–14 μm long, 3–5 μm wide (x̄= 9.5 × 4 μm, n = 20), hyaline to pale brown, smooth-walled. ***Conidia*** solitary, acropleurogenous, helicoid, rounded at tip, 16–21 μm diam conidial filament 1.5–4 μm wide (x̄ = 19 × 3 μm, n = 30), 127–175 μm long (x̄ = 144 μm, n = 30), indistinctly septate, coiled 3^1^/_2_–3^3^/_4_ times, becoming uncoiled in water, guttulate, hyaline, smooth-walled.

**Figure 3.**
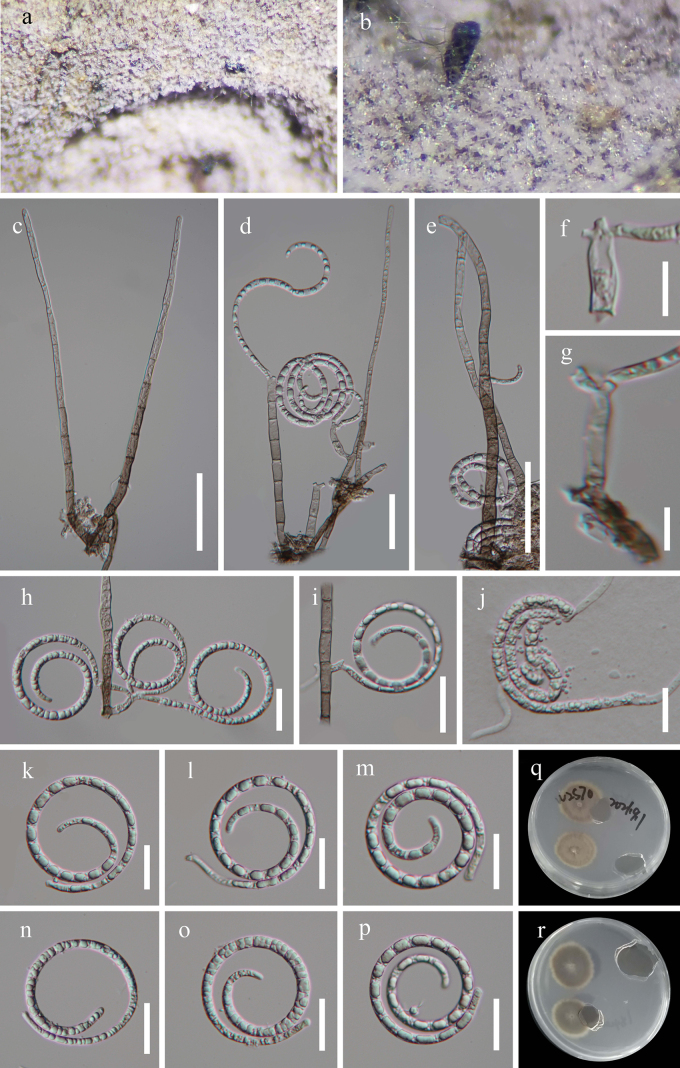
*Tubeufiahainanensis* (HKAS 125884, holotype) **a, b** colonies on the host surface **c–e** conidiophores, conidiogenous cells and conidia **f–h** conidiogenous cells bearing conidia **j–o** conidia **i** germinated conidium **p, q** colonies on PDA, **p** from above **q** from below. Scale bars: 20 μm (**c–e, i–l**); 10 μm (**f–h, m–o**).

##### Culture characteristics.

Conidia germinating on water agar and producing germ tubes within 8 h. Colonies on PDA circular with umbonate surface and undulate edge. Growth rate 43 mm diam in 50 days at 25 °C, with a pale brown surface.

##### Material examined.

China, Hainan Province, Haikou City, Xiuying District, Ecological leisure trail, 20°01′N, 110°25′E, on decaying wood in terrestrial habitat, 10 August 2021, Jian Ma, HK1 (HKAS 125884, holotype; GZAAS 22–2015, isotype), ex-type living cultures CGMCC, GZCC 22–2015; *Ibid.*, HK1-2 (HKAS 125883, paratype), living culture GZCC 23–0589.

##### Notes.

*Tubeufiahainanensis* resembles *T.parvispora* morphologically, with solitary, acropleurogenous, hyaline, helicoid conidia. However, *Tubeufiahainanensis* can be distinguished from *T.parvispora* by its unbranched conidiophores (Lu et al. 2018b). *Tubeufiahainanensis* formed a sister clade to Tubeufiaceae (BBC 3512) with 85% ML/0.99 PP supports (Fig. 1), and the phylogeny indicated that it is distinct species.

#### ﻿ Tubeufia muriformis

Taxon classificationFungiTubeufialesTubeufiaceae

J. Ma & Y.Z. Lu sp. nov.

5AA521B9-F1AA-5894-ACC0-8BABCAAF3C8C

900506

Facesoffungi Number: FoF14267

Fig. 4

##### Etymology.

The epithet ‘‘*muriformis*’’ refers to the multi-septate conidia of this taxon.

##### Holotype.

HKAS 128853.

##### Description.

***Saprobic*** on decaying bamboo in a terrestrial habitat. ***Sexual morph*** Undetermined. ***Asexual morph*** Hyphomycetous, helicosporous. ***Colonies*** on natural substrate superficial, effuse, gregarious, white. ***Mycelium*** superficial, partly immersed, hyaline to pale brown, septate, branched hyphae, smooth, with masses of crowded, glistening conidia. ***Conidiophores*** macronematous, mononematous, straight or flexuous, simple, cylindrical, branched or unbranched, indistinctly septate, 13–36 μm long, 3.5–7.5 μm wide, hyaline, smooth-walled. ***Conidiogenous cells*** holoblastic, monoblastic, integrated, sympodial, terminal, cylindrical, truncate at apex after conidial secession, hyaline, smooth-walled. ***Conidia*** solitary, acrogenous, muriform, curved, 23–25 μm diam and conidial filament 11–15 μm wide (x̄ = 24 × 13 μm, n = 20), 16.5–58.5 μm long (x̄ = 49 μm, n = 20), composed of two rows of cells with pale, multi-septate, apical cells cylindrical, basal cells truncate, constricted at septae, tapering toward base and top of conidia, coiled ^1^/_4_–1 times, not becoming loose in water, guttulate, hyaline to pale brown, thick-walled, smooth-walled.

##### Culture characteristics.

Conidia germinating water agar and producing germ tubes within 12 h. Growth on PDA with a circular shape and umbonate surface and entire edge. Growth rate 42 mm diameter in 40 days at 25 °C with a pale brown surface.

**Figure 4.**
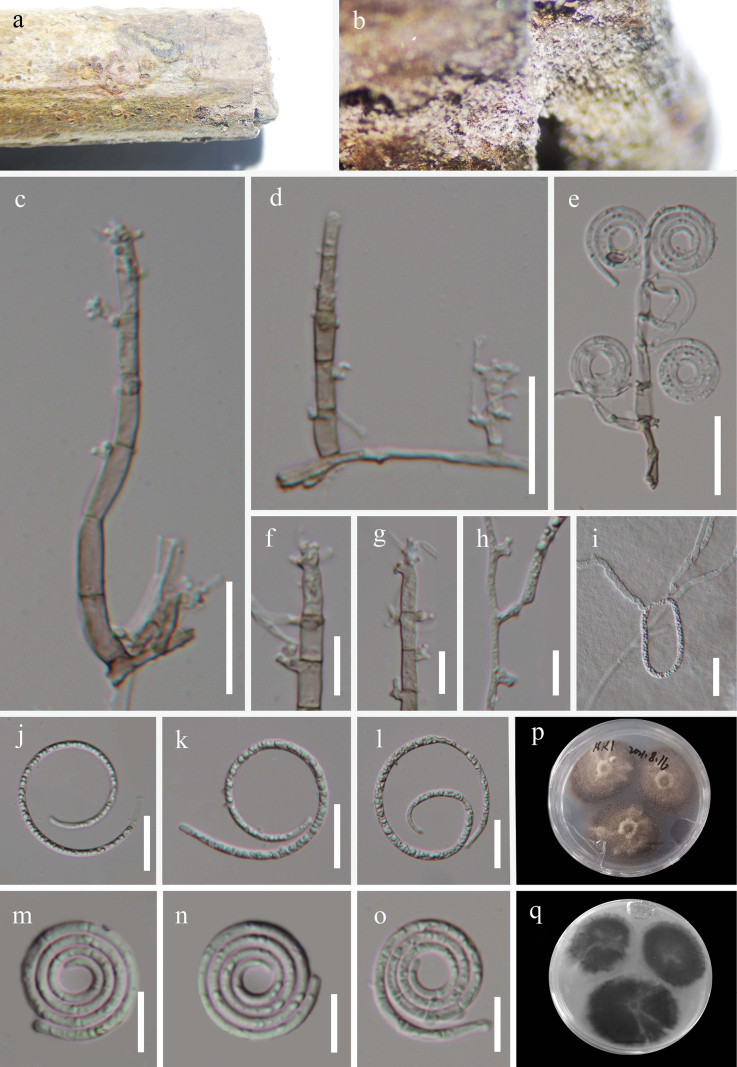
*Tubeufiamuriformis* (HKAS 128853, holotype) **a, b** colonies on the host surface **c–e** conidiophores, conidiogenous cells and conidia **d, f–i** conidiogenous cells **k–o** conidia **j** germinated conidium **p, q** colonies on PDA, **p** from above **q** from below. Scale bars: 20 μm (**c–f, j–k**); 10 μm (**g–i, l–o**).

##### Material examined.

China, Guizhou Province, Qianxinan Prefecture, Xianheping National Forest Park, 24°97′N, 105°63′E, on submerged decaying wood in a freshwater stream, 16 March 2022, Jian Ma, XHP38 (HKAS 128853, holotype; GZAAS 22-2039, isotype), ex-type living cultures CGMCC, GZCC 22–2039; *Ibid.*, XHP64 (GZAAS 23–0594, paratype), living culture GZCC 23–0591.

##### Notes.

*Tubeufiamuriformis* shares morphological similarities with *Xenosporiumhelicominum*, characterized by the presence of mononematous, straight or flexuous conidiophores, monoblastic, terminal, cylindrical conidiogenous cells, and muriform, curved, hyaline to pale-brown conidia. However, *T.muriformis* can be distinguished from *X.helicominum* with its branched conidiophores and larger conidia (23–25 × 11–15 μm vs. 14–16.5 × 5.5–6.5 µm; Zhao et al. 2007). Moreover, *T.muriformis* differs from other *Tubeufia* species in that it has muriform, curved conidia (Lu et al. 2018b).

The phylogenetic analysis indicated that *Tubeufiamuriformis* formed a closely related clade with *T.bambusicola*, supported by ML bootstrap value of 100% and PP of 1.00. This phylogenetic relationship confirms that *Tubeufiamuriformis* and *T.bambusicola* are distinct species, as demonstrated by the phylogenetic tree.

#### ﻿ Tubeufia cocois

Taxon classificationFungiTubeufialesTubeufiaceae

X.G. Tian & Tibpromma et al. Journal of Fungi 8: 22 (2021).

86FDC482-A653-5E51-B31C-A04A6B026739

555070

Facesoffungi Number: FoF10576

Fig. 5

##### Description.

***Saprobic*** on submerged decaying wood in a freshwater stream. ***Sexual morph*** Undetermined. ***Asexual morph*** Hyphomycetous, helicosporous. ***Colonies*** on natural substrate superficial, effuse, gregarious, white. ***Mycelium*** superficial and partly immersed, hyaline, septate, branched hyphae, smooth, with glistening conidia. ***Conidiophores*** macronematous, mononematous, straight or slightly flexuous, cylindrical, unbranched, septate, 33–85 μm long, 5–7.5 μm wide (x̄ = 48.5 × 6 μm, n = 20), the lower part pale brown and the upper part hyaline, smooth-walled. ***Conidiogenous cells*** holoblastic, polyblastic, integrated, sympodial, terminal, cylindrical, denticulate, with a tooth-like protrusion, 1.5–4 μm long, 1.5–2.5 μm wide, truncate at apex after conidial secession, 4.5–10.5 μm long, 4.5–6 μm wide (x̄ = 8.5 × 5.5 μm, n = 20), hyaline, smooth-walled. ***Conidia*** solitary, acropleurogenous, helicoid, rounded at tip, 23–29 μm diam and conidial filament 4–6.5 μm wide (x̄ = 26 × 5.5 μm, n = 30), 100.5–138 μm long (x̄ = 118 μm, n = 25), indistinctly septate, coiled 2–2^1^/_2_ times, not becoming loose in water, guttulate, hyaline, smooth-walled.

**Figure 5.**
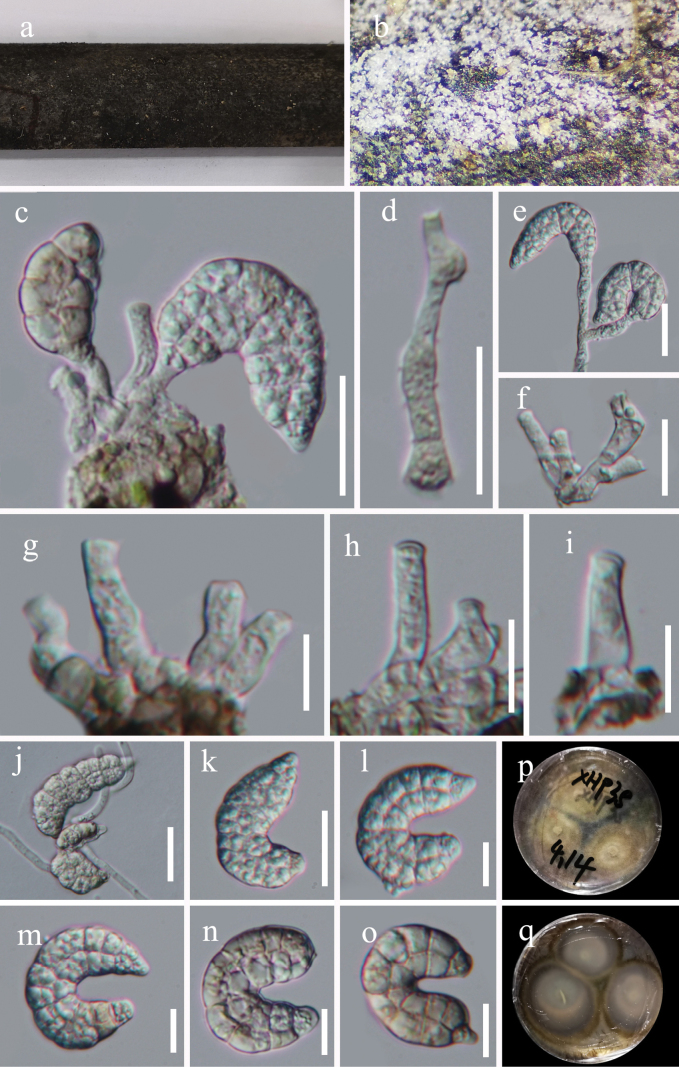
*Tubeufiacocois* (GZAAS 22–2038) **a, b** colonies on the host surface **c–f** conidiophores and conidiogenous cells **g–j** conidiogenous cells **l–q** conidia **k** germinated conidium **r, s** colonies on PDA, **r** from above **s** from below. Scale bars: 20 μm (**c, d, f**); 10 μm (**e, g–q**).

##### Culture characteristics.

Conidia germinating on water agar and producing germ tubes within 8 h. Colonies on PDA circular with flat surface and undulate edge. Growth rate 26 mm diameter in 35 days at 25 °C, with a dark brown to black surface.

##### Material examined.

China, Hainan Province, Qiongzhong Li and Miao Autonomous County, Baihualing Rainforest cultural tourism area, 18°98′N, 109°82′E, on rotting wood in a freshwater stream, 29 December 2021, Jian Ma, BH5 (GZAAS 22–2038), living culture GZCC 22–2038.

##### Notes.

Tian et al. (2022) introduced *Tubeufiacocois* from the dead leaves of *Cocosnucifera* in Thailand. Our newly isolated strain (GZCC 22–2038) clusters with *T.cocois* (MFLUCC 22–0001) with 93% ML/1.00 PP support. Morphologically, our new isolate shares the same morphological characteristics with the holotype (MFLU 21–0192) of *T.cocois*, thus we identified our new isolate as *T.cocois*. This is the first discovery of this species in a freshwater habitat and the first discovery of this species in China.

## ﻿Discussion

In this study, a total of seven helicosporous hyphomycetous taxa were collected from the southern Chinese provinces of Guizhou and Hainan. By utilizing a combination of multigene phylogenetic analysis and morphological evidence, three previously unknown species were characterized and designated as *Tubeufiaguttulata*, *T.hainanensis*, and *T.muriformis*. Additionally, an additional taxon, *T.cocois*, was documented for the first time in this study.

*Tubeufia* is the largest genus within the family Tubeufiaceae. Currently, this genus contains 59 species (Lu et al. 2018b, 2023; Rajeshkumar et al. 2019; Boonmee et al. 2021; Li et al. 2022; Tian et al. 2022), including the newly introduced species in this study, of which 29 are found in freshwater habitat, 24 in terrestrial habitats, and six from both freshwater and terrestrial habitats (Barr 1979; Rossman 1987; Ho et al. 2002; Tsui and Berbee 2006; Tsui et al. 2007; Zhao et al. 2007; Boonmee et al. 2011, 2014; Chaiwan et al. 2017; Dai et al. 2017; Doilom et al. 2017; Lu et al. 2017, 2018b, 2022, 2023; Tian et al. 2022). Among them, 39 species produce a helicosporous conidial state.

It should be noted that the morphological features of helicosporous fungi belonging to the genus *Tubeufia* exhibit distinct differences compared to other helicosporous genera. Summarizing the morphological characteristics of *Tubeufia* at the genus level is challenging due to the absence of similarity in conidiophores and conidia among its species. Such as the morphology of the newly discovered species *Tubeufiamuriformis* resembles *Xenosporium* rather than *Tubeufia* (Goos 1990; Zhao et al. 2007). This discovery expands the asexual morphological characteristics of the genus *Tubeufia*, which is distinguished by conidia that are curved dorsoventrally. Lu et al. (2018b) reported dictyosporous conidia in *Tubeufia*, while Rajeshkumar et al. (2019) discovered a new dictyosporous asexual morph, indicating that this genus may contain many undiscovered species. In addition, the sexual morphs of *Tubeufia* exhibit considerable diversity, indicating a rapid evolutionary rate within this genus. The wide range of morphological variations observed may be related to the adaptation of species to environmental changes. Given the recent research progress in uncovering new structurally active compounds in *Tubeufia* species, there is a compelling need for further investigations into the taxonomy and secondary metabolites of this genus (Chen et al. 2020; Zeng et al. 2022; Qian et al. 2023).

## Supplementary Material

XML Treatment for Tubeufia guttulata

XML Treatment for Tubeufia hainanensis

XML Treatment for Tubeufia muriformis

XML Treatment for Tubeufia cocois

## ﻿Additional information

### Author ORCIDs

Jian Ma https://orcid.org/0009-0008-1291-640X

Li-Juan Zhang https://orcid.org/0000-0002-3234-6757

Saranyaphat Boonmee https://orcid.org/0000-0001-5202-2955

Xing-Juan Xiao https://orcid.org/0009-0003-8769-4534

Ning-Guo Liu https://orcid.org/0000-0002-9169-2350

Yuan-Pin Xiao https://orcid.org/0000-0003-1730-3545

Zong-Long Luo https://orcid.org/0000-0001-7307-4885

Yong-Zhong Lu https://orcid.org/0000-0002-1033-5782

### Data availability

All of the data that support the findings of this study are available in the main text.
